# A Pocket Text-Book of Obstetrics

**Published:** 1901-04

**Authors:** 


A Pocket Text-Book of Obstetrics. By David J. Evans, M. D , Lecturer on Obstetrics and Diseases of Infancy in McGill University Faculty of Medicine, Montreal. 12mo, 409 pages, with 409 illustrations, partly in colors. Lea's Series of Pocket Text-Books. Edited by Bern B. Gallaudet, M. D. Philadelphia and New York: Lea Brothers & Co. 1901. [Cloth, $1.75, net; full flexible leather, $2.25, net.]

A satisfactory work on obstetrics can hardly be condensed into the limits of a volume like the present, yet with this feeling in mind the reviewer is forced to confess that the author has succeeded admirably. Much is, of course, omitted from necessity, much is abbreviated, sometimes almost to obscurity, but on the whole the book is a good one, and in the practical part of the subject most excellent, because thoroughly modern. M. J. F.

